# Work ability and productivity in patients with diabetic foot

**DOI:** 10.6061/clinics/2019/e421

**Published:** 2019-03-19

**Authors:** Helga dos Santos Cabeceira, Diba Maria Sebba Tosta de Souza, Yara Juliano, Daniela Francescato Veiga

**Affiliations:** I Mestrado Profissional em Ciencias Aplicadas a Saude, Universidade do Vale do Sapucai (UNIVAS), Pouso Alegre, MG, BR; II Departamento de Enfermagem, Mestrado Profissional em Ciencias Aplicadas a Saude, Universidade do Vale do Sapucai (UNIVAS), Pouso Alegre, MG, BR; III Departamento de Bioestatistica, Universidade do Vale do Sapucai (UNIVAS), Pouso Alegre, MG, BR; IV Divisao de Cirurgia Plastica, Mestrado Profissional em Ciencias Aplicadas a Saude, Universidade do Vale do Sapucai (UNIVAS), Pouso Alegre, MG, BR

**Keywords:** Diabetes Mellitus, Diabetic Foot, Efficiency, Absenteeism, Work

## Abstract

**OBJECTIVE:**

To assess work ability and productivity in patients with diabetic foot.

**METHODS:**

This investigation was a cross-sectional controlled study. A total of 117 individuals were selected from March to June 2014 and allocated to group A (patients without diabetes, n=43), group B (diabetes patients without foot ulcers, n=43), or group C (patients with diabetic foot, n=31). Two validated instruments, the Work Limitations Questionnaire (WLQ) and the Work Productivity and Activity Impairment Questionnaire General Health v2.0 (WPAI-GH), were used to assess work ability and productivity.

**RESULTS:**

The groups were homogeneous regarding age and sex; however, patients in group C had a lower education level than the other participants (*p*=0.006). The median WLQ scores for groups A, B, and C were 0.0121, 0.0146, and 0.0852, respectively (*p*<0.0001). The WPAI-GH scores revealed a mean productivity loss of 20% for groups A and B and 100% for group C (*p*<0.0001).

**CONCLUSIONS:**

Patients with diabetic foot showed decreased work ability and productivity.

## INTRODUCTION

Diabetes mellitus is a metabolic dysfunction of multiple etiologies, characterized by chronic hyperglycemia resulting from a deficiency in insulin secretion or the inability of insulin to exert its normal effects 1. This condition requires lifelong medical care. Diabetes significantly increases the risk of cardiovascular disease and is the most common cause of nontraumatic lower limb amputation, visual loss, and end-stage renal disease 1. Diabetes and its related complications are becoming the most significant cause of morbidity and mortality in the global population. It has been estimated that over 642 million people will have diabetes worldwide by 2040 2.

Tissue dysfunction aggravated by trauma combines with diabetes-related vulnerability to infections to result in complex clinical problems generally classified as diabetic foot; this condition is one of the most serious complications of diabetes mellitus and a risk factor for lower limb amputation 3. Diabetic foot is associated with the presence of infection, ulceration, or destruction of deep tissues combined with various degrees of peripheral vascular disease and neurological abnormalities 2, including neuropathy, which can affect sensory, motor and autonomic nerves 3.

Diabetes mellitus is highly prevalent throughout the world and may result in reduced work ability, causing patients to withdraw from work. To the best of our knowledge, this investigation is the first study aiming to assess work ability and productivity in Brazilian patients with diabetic foot.

## MATERIALS AND METHODS

This investigation was a controlled cross-sectional study. The study protocol was approved by the Research Ethics Committee of the University of Vale do Sapucaí [Certificate of Filing for Ethical Approval (CAAE) number 25563513.9.0000.5102; approval number 482342]. The study was performed in accordance with the ethical standards of the 1964 Declaration of Helsinki and its subsequent amendments and with Resolution 466/12 of the Brazilian National Health Council (CNS) on research involving human beings. Written informed consent was obtained from all patients prior to their inclusion in the study, and the subjects were aware that they were free to leave the study at any time. Patient autonomy, privacy, and anonymity were assured.

Patients from outpatient clinics of the Samuel Libânio General Hospital and Municipal Diabetes Education Centers (CEMED) in Pouso Alegre (Minas Gerais, Brazil) were selected from March to June 2014 to participate in the study.

A total of 117 individuals were enrolled and allocated to group A (patients without diabetes, n=43), group B (diabetes patients without foot ulcers, n=43), or group C (diabetes patients with foot ulcers, n=31).

Patients of both sexes who were between 30 and 60 years old and had type 1 or 2 diabetes mellitus were eligible for the study. There was no restriction on the nature of the patients' work (i.e., formal or informal). Individuals with gestational diabetes were not included in the sample.

Patients with diabetic foot ulcers were included in group C, and diabetes patients without foot ulcers were included in group B. Individuals without diabetes mellitus and with similar demographic characteristics to those in groups B and C were selected for group A (controls).

For the patients in group C, diabetic foot ulcers were graded according to the Wagner classification system 4, which is based on the extent, intensity and depth of ulcerations and the presence of infection, osteomyelitis, and necrosis.

The Brazilian versions of two validated instruments, namely, the Work Limitations Questionnaire (WLQ) 5 and Work Productivity and Activity Impairment Questionnaire General Health v2.0 (WPAI-GH) 6, were used to assess the work ability and productivity of participants. All interviews were conducted by the same researcher (HSC).

As described previously 5, the WLQ measures the impact of health conditions on work performance and estimates the consequent decline in productivity. Presenteeism is assessed by the degree of difficulty the worker faces in performing specific tasks required at work, as defined in previous studies 5,7. According to the literature 5,7,8, the purpose of the WLQ is to increase the quantity and quality of information about disability and loss of productivity at work. This questionnaire is an easy-to-use 25-item instrument that takes an average of five to ten minutes to complete 8. The 25 items are grouped into four subscales: time management, physical demands, mental-interpersonal demands, and output demands. The time management subscale assesses difficulties in handling time and scheduling tasks. The physical demands subscale evaluates the ability to perform work tasks that require bodily strength. The mental-interpersonal demands subscale addresses difficulties in performing cognitively demanding tasks at work and in social interactions on the job. The output demands subscale refers to difficulties with work quantity and quality 5,7. The scores on each subscale range from 0 (none of the time) to 100 (all of the time), indicating the percentage of time that the individual was limited in performing work tasks within the last two weeks. The WLQ index is computed as the weighted sum of the subscale scores 5,7.

As reported in previous studies 6,9, the Brazilian version of the WPAI-GH is a useful instrument that measures the impact of health conditions on the productivity of a working population. The WPAI-GH is composed of six items assessing deficits in work productivity and activities in the past seven days. This questionnaire is scored on the basis of four indicators: the percentage of time lost from work due to health conditions (absenteeism), the percent reduction in work effectiveness due to health conditions (presenteeism), the percentage of overall work productivity lost due to health conditions (absenteeism and presenteeism), and the percent decline in activities of daily living (ADL) outside of work due to health conditions, with higher scores indicating longer absence due to sickness or lower productivity 10.

### Statistical analysis

The Kruskal-Wallis test was used to compare the study variables among the three groups, and the chi-squared test was used to compare categorical variables between groups. The level of significance was set at an alpha of 0.05 (*p*<0.05) for all tests.

## RESULTS

The occupations of the study participants included general assistant, housekeeper, seamstress, construction worker, truck driver, freelancer, lawyer, physician, self-employed worker, nurse, administrative assistant, receptionist, glazier, retailer, upholsterer, pharmacist, journalist, banker, teacher, and business person.

The sociodemographic characteristics of the patients allocated to the three groups are shown in Table 1. The mean duration of diabetic foot was 11±23.3 months (median, 14.5) for patients in group C.

The WLQ scores indicated a significant difference in work limitation among groups. Patients in group C reported higher WLQ scores, indicating a greater decline in productivity, than patients in groups A and B (Table 2).

The distribution of patients in group C by severity of foot ulcers, according to the Wagner classification system, is depicted in Table 3. Most patients were classified as grade 1, showing superficial fungal infection or mild bacterial infection.

The WPAI-GH percent values are listed in Table 4. Patients in group C had elevated WPAI-GH values compared with those in groups A and B.

## DISCUSSION

Chronic diseases and their treatments may cause symptoms that impair individual performance at work. A recent study on the impact of diabetes and diabetes-related complications on productive activities among older Europeans showed that the presence of diabetes increased the probability of being afraid that health would limit work before retirement by nearly 11% (adjusted for clinical complications) and reduced the likelihood of being a formal volunteer by 2.7% (adjusted for mobility problems) 11.

Diabetes mellitus is more common in men than in women; in fact, the number of people with diabetes worldwide is projected to increase from 198 million men and 184 million women in 2013 to 303 million men and 288 million women in 2035 12. The present study confirms this trend, since most patients in group C were men; however, no significant difference in gender was found among groups.

In both sexes, diabetes is marked by the chronicity of the disease and associated complications, which are often present at different times in patients' lives, including when individuals are engaged in productive activities in the labor market. A study carried out in Canada found that diabetes mellitus is associated with various occupational health outcomes, including work-related injury, loss of work productivity, and occupation type 13.

Foot-care practice is usually poor among patients with diabetes 14. Low education levels may limit or patients from acquiring information on prevention and appropriate self-care regarding the condition. In this study, patients with diabetic foot (group C) had lower education levels than patients in groups A and B, which is in agreement with a previous study on the risk factors for the development of diabetic foot ulcerations 15. It is important to offer multidisciplinary educational interventions to both patients and their families. A previous study suggested that improvements in physical working conditions and a reduction in smoking, especially among employees with low education, may markedly reduce differences across education levels in the amount of work missed due to sickness (16).

According to the Wagner classification system, most patients (54.8%) in group C had foot ulcers classified as grade 1 (infections and superficial ulcers), followed by 25.8% of patients with foot ulcers classified as grade 2 (deep ulcers). However, even with 80% of patients in group C falling within the two lowest Wagner grades, the percentages of absenteeism, presenteeism, and decline in ADL were significantly higher in group C than in groups A and B (see Table 4).

The results of this study revealed reduced work ability among patients with diabetic foot ulcers, indicating the need for systematic application of risk-assessment tools for foot ulcerations and injuries in workers with diabetes. The commitment of employers to the health of their employees is also necessary. It is essential for employers to promote education and training programs on the risk of diabetic foot and to provide managers with adequate information on preventive screening, thus promoting the health of employees, preventing a decline in productivity associated with health complications, reducing employers' health-related costs and health-care costs, and improving productivity.

Patient scores on the time management subscale of the WLQ showed that the patients with diabetic foot (group C) had increased difficulty managing time and scheduling tasks compared with the controls and patients with diabetes but without ulcerations. Patients in group C reported increased scores on the physical demands subscale, which is associated with increased difficulty in performing tasks that require bodily strength, movement, endurance, coordination, and flexibility. Patients in group C also presented limitations in performing cognitive tasks and interacting with people at work, as well as a decline in their ability to complete work on time in the quantity and quality required. With regard to absenteeism, group C reported the greatest time lost from work due to health conditions, followed by patients with diabetes but without foot ulcers (group B). Stress, poor mental health, poor quality of life, and a history of neuropathy have been identified as factors associated with increased presenteeism in people with type 2 diabetes mellitus 17. A recent prospective survey study with health-care industry workers from two integrated health systems indicated that the presence of diabetes mellitus was associated with increased rates of lost productivity, elevated indirect costs for employers, and increased use of health-care resources 18.

Other authors have studied productivity and work ability among patients with other chronic conditions 8,. A study comparing employees with and without depression found that the work performance of depressed employees was negatively affected by psychosocial stressors including high psychological demands, limited job control, and insufficient social support 8. The amount of time with work limitations was related to time management, physical tasks, mental-interpersonal job tasks, and output tasks 8.

In a study on the impact of weight loss on work absenteeism and presenteeism among 1030 overweight or obese participants who completed a survey at baseline and 12 months after a weight-loss intervention, no significant correlation was observed between weight loss and work productivity 19.

Chronic obstructive pulmonary disease (COPD) is associated with extensive use of health care resources, representing a significant socioeconomic burden and frequently resulting in work impairment and productivity loss in the working-age population 20.

In a retrospective study on health-related work productivity and work impairment among employed adults with osteoarthritis, 70% of patients reported presenteeism, whereas 10% reported absenteeism 21. Osteoarthritis patients who reported presenteeism also reported greater use of medication, lower health-related quality of life (HRQoL) in both the mental and physical components of the 36-Item Short-Form Health Survey (SF-36), and higher depression severity scores than other study participants 21.

Another study indicated that breast reduction improved work capacity and productivity in women with breast hypertrophy 22. Thus, breast reduction may be considered by managers of public and corporate health institutions as a means of providing direct quality-of-life benefits for this population and indirect economic benefits for the production sector 22.

Presenteeism is the percent reduction in work effectiveness due to health conditions 10. Presenteeism occurs when the worker is physically present at the workplace but is totally or partially affected by physical or psychological factors that reduce his or her degree of commitment or effectiveness in work tasks 23.

Absenteeism is the percentage of work time lost due to health conditions 10 and is also an important indicator of a worker's health state 24. Significant associations of hypoglycemia severity with HRQoL, productivity loss, use of health care resources, and treatment costs have been observed among patients with type 2 diabetes mellitus 25. Hypoglycemia severity seems to be related to reduced HRQoL and productivity loss, leading to increased direct and indirect health care costs 25.

The implementation of education and training programs on diabetic foot risks and preventive measures in the workplace would provide managers with adequate information to motivate healthy behavior among employees, thereby promoting health in the workplace, preventing a decline in productivity associated with health complications, and reducing employer health-related costs and health-care costs.

Most diabetic foot infections start with a sore. Once the infection occurs, the risk of hospitalization and amputation rises dramatically. Early identification of potential risk factors for infection combined with prompt treatment may optimize the outcome and prevent amputation 26. Foot screening is an important part of diabetes care, significantly decreasing morbidity, loss of function, and mortality from diabetic foot complications. However, foot screening is often neglected. A study designed to educate health professionals at a primary-care clinic 26 resulted in an increase in diabetic foot screening practices from 9% to 69% after the first cycle of quality improvement, with a significant rise in the number of diabetes patients screened 26. Education should be directed at patients, caregivers and health professionals so that they can effectively provide appropriate care and information 27. Patient education includes information on diabetes care, foot care, and the use of appropriate footwear. Because patients tend to have poor foot hygiene, an annual foot screening is important for people diagnosed with diabetes. However, prolonged and sustained government intervention is necessary to provide education and screening on a national scale 27.

The quality of life of patients with diabetic foot ulcers reflects their living conditions and the health-care systems of their respective countries 28. Sociodemographic factors and clinical characteristics should be considered in nursing care and may have an impact not only on the management of diabetic foot but also on the recognition of the patient as an individual with unique experiences in the physical, psychosocial, and environmental domains 28.

The lack of preventive measures for diabetic foot may negatively impact patients' quality of life and, consequently, their work activities 29. Prevention is the most important strategy, and education is key. A previous qualitative study was structured around technology, current health practices related to diabetic foot care, and the use of mobile health (mHealth) devices to prevent and monitor diabetes patients' foot ulcers 29. Patients with diabetic foot ulcers expressed interest in mHealth for preventing foot ulcers and monitoring diabetic foot care, although some participants were not frequent users of technology 29. The study showed that mHealth has the potential to improve patient outcomes 29.

The present study showed that patients with diabetic foot had a reduced ability to work in addition to difficulty in managing time on the job, performing work tasks that required bodily strength and endurance, and maintaining productivity. This work also highlighted the difficulty these patients faced with cognitively demanding tasks, information management, performance in interpersonal interactions, and capacity to perform the expected quantity and quality of work within an appropriate time frame. Thus, patients with diabetic foot showed reduced efficiency and increased time lost from work due to their health conditions, resulting in losses in work productivity.

## CONCLUSIONS

Patients with diabetic foot have an impaired ability to work, and they face difficulty in meeting deadlines and performing tasks that require bodily strength and endurance. Such patients also have difficulty performing activities that require mental effort, information management, and interpersonal interactions, and they show a diminished ability to deliver the required quantity and quality of work in a timely manner.

## AUTHOR CONTRIBUTIONS

Cabeceira HS contributed to the design of the study the acquisition, analysis and interpretation of the data, and the manuscript preparation. Souza DMST participated in the conception and design of the study, the analysis and interpretation of the data, and the preparation and critical revision of the manuscript with respect to important intellectual content. Juliano Y contributed to the design of the study, performed the statistical analysis, and revised the manuscript critically for important intellectual content. Veiga DF participated in the conception and design of the study, the analysis and interpretation of the data, and the critical revision of the manuscript for important intellectual content. All authors approved the final version of the manuscript and take public responsibility for the appropriate portions of the content.

## Figures and Tables

**Table 1:** Sociodemographic characteristics of the study participants.

Variables		Group A (n=43)	Group B (n=43)	Group C (n=31)	*p*-value
Age (years)	Mean	48	48	52	0.085^a^
	Median	53	49	53	
Sex	Male	21	23	20	0.401^b^
	Female	22	20	11	
Level of education	Incomplete primary education	6	6	15	0.006^b,*^
	Incomplete high school education	22	19	5	
	Complete high school education	5	7	5	
	Higher education	9	11	6	

^a^Kruskal-Wallis test; ^b^chi-squared test; *statistical significance (*p*<0.05).

Group A, controls; Group B, diabetes patients without foot ulcers; Group C, diabetes patients with foot ulcers.

**Table 2:** WLQ subscale scores for the different study groups.

WLQ subscales	Group A (n=43)		Group B (n=43)		Group C (n=31)		*p*-value	Multiple comparisons
	Mean	Median	Mean	Median	Mean	Median		
Time management	3.14	0	12.44	5.00	42.10	35.00	<0.0001*	C>B,A
Physical demands	8.53	4.17	14.15	4.17	33.74	33.33	<0.0001*	C>B,A
Mental-interpersonal demands	6.65	5.56	11.95	8.33	24.37	19.44	<0.0001*	C>B,A
Output demands	3.49	0	10.58	5.00	37.42	35.00	<0.0001*	C>B,A
WLQ index	0.0147	0.0121	0.0338	0.0146	0.0954	0.0852	<0.0001*	C>B,A

Kruskal-Wallis test; *statistical significance (*p*<0.05).

A or Group A, controls; B or Group B, diabetes patients without foot ulcers; C or Group C, diabetes patients with foot ulcers.

**Table 3:** Distribution of patients with diabetic foot according to the Wagner classification system.

Diabetic foot severity	Men (N)	Women (N)	Total (N)	*p*-value
Grade 1	12	5	17	
Grade 2	4	4	8	
Grade 3	3	0	3	0.1442
Grade 4	0	2	2	
Grade 5	1	0	1	
**Total (N)**	**20**	**11**	**31**	

Chi-squared test (*p*<0.05).

**Table 4:** WPAI-GH percent values for the three groups.

WPAI-GH indicators		Group A	Group B	Group C	*p*-value	Multiple comparisons
Absenteeism (%)	Mean	0.1	4.9	64.1		C>A,B
	Median	0	0	100	<0.0001	
Presenteeism (%)	Mean	20.5	30.4	76.8		C>A,B
	Median	20.0	20.0	100	<0.0001	
Absenteeism + Presenteeism (%)	Mean	20.5	31.0	80.6		C>A,B
	Median	20.0	20.0	100	<0.0001	
Decline in ADL (%)	Mean	24.4	28.1	60.6		C>A,B
	Median	20.0	20.0	70.0	<0.0001	

ADL, activities of daily living; A or Group A, controls; B or Group B, diabetes patients without foot ulcers; C or Group C, diabetes patients with foot ulcers.

Kruskal-Wallis test (*p*<0.05).
